# Establishment of a PCR Assay for the Detection and Discrimination of Authentic Cordyceps and Adulterant Species in Food and Herbal Medicines

**DOI:** 10.3390/molecules23081932

**Published:** 2018-08-02

**Authors:** Byeong Cheol Moon, Wook Jin Kim, Inkyu Park, Gi-Ho Sung, Pureum Noh

**Affiliations:** 1 Division of Herbal Medicine Research, Korea Institute of Oriental Medicine, Daejeon 34054, Korea; ukgene@kiom.re.kr (W.J.K.); pik6885@kiom.re.kr (I.P.); pureum322@kiom.re.kr (P.N.); 2 Institute for Bio-Medical Convergence, College of Medicine, Catholic Kwandong University, Incheon 22711, Korea; sung97330@gmail.com; 3 Department of Microbiology, College of Medicine, Catholic Kwandong University, Gangneung 25601, Korea

**Keywords:** *Ophiocordyceps sinensis*, *Cordyceps militaris*, sequence characterized amplified region (SCAR) marker, real-time PCR, molecular authentication

## Abstract

Accurate detection and differentiation of adulterants in food ingredients and herbal medicines are crucial for the safety and basic quality control of these products. *Ophiocordyceps sinensis* is described as the only fungal source for the authentic medicinal ingredient used in the herbal medicine “Cordyceps”, and two other fungal species, *Cordyceps militaris* and *Isaria tenuipes*, are the authentic fungal sources for food ingredients in Korea. However, substitution of these three species, and adulteration of herbal material and dietary supplements originating from *Cordyceps pruinosa* or *Isaria cicadae*, seriously affects the safety and reduces the therapeutic efficacy of these products. Distinguishing between these species based on their morphological features is very difficult, especially in commercially processed products. In this study, we employed DNA barcode-based species-specific sequence characterized amplified region (SCAR) markers to discriminate authentic herbal Cordyceps medicines and Cordyceps-derived dietary supplements from related but inauthentic species. The reliable authentication tool exploited the internal transcribed spacer (ITS) region of a nuclear ribosomal RNA gene (nrDNA). We used comparative nrDNA-ITS sequence analysis of the five fungal species to design two sets of SCAR markers. Furthermore, we used a set of species-specific SCAR markers to establish a real-time polymerase chain reaction (PCR) assay for the detection of species, contamination, and degree of adulteration. We confirmed the discriminability and reproducibility of the SCAR marker analysis and the real-time PCR assay using commercially processed food ingredients and herbal medicines. The developed SCAR markers may be used to efficiently differentiate authentic material from their related adulterants on a species level. The ITS-based SCAR markers and the real-time PCR assay constitute a useful genetic tool for preventing the adulteration of Cordyceps and Cordyceps-related dietary supplements.

## 1. Introduction

*Ophiocordyceps sinensis* (Berk.) G.H. Sung, J.M. Sung, Hywel-Jones and Spatafora (syn. *Cordyceps sinensis* (Berk.) Sacc.), a constituent of the traditional herbal medicine Cordyceps, is an entomogenous and well-known medicinal fungus that belongs to the family Ophiocordycipitaceae (division Ascomycota) [1]. According to the Korean and Chinese Pharmacopoeia, the fungal source of the herbal medicine ‘Cordyceps’, namely *Dong Chung Ha Cho* in Korean and *Dong Chong Xia Cao* in Chinese, is described as originating only from the species *O. sinensis* [2]. However, several other fungal species, *Isaria cicadae* Miq. (syn. *Cordyceps cicadae* (Miq.) Massee or *Paecilomyces cicadae*), *Cordyceps militaris* (L.) Fr., *Cordyceps pruinosa* Petch., *Cordyceps gunnii* Berk., *Ophiocordyceps robertsii* (Hook.) Berk., and *Ophiocordyceps ophioglossoides* (J.F. Gmel.) Fr., are repeatedly used as adulterants of authentic Cordyceps [1,3]. Furthermore, only two species, *C. militaris* and *Isaria tenuipes* Peck (syn. *Cordyceps polyarthra* or *Paecilomyces tenuipes*), are described as authentic sources of food ingredients in the Korean market [4]. Hence, only *O. sinensis*-derived material can be used for medicinal purposes in Korean traditional medicine, whereas only the material derived from *C. militaris* or *I. tenuipes* can be used as food in Korea [2,4]. However, Cordyceps-related species are frequently substituted for one another in herbal and food material because of similar morphological features and confusing homonymous names; furthermore, Cordyceps derived from *O. sinensis* is very expensive (ca. 54 USD per g) [5]. *C. pruinosa* Petch. and *I. cicadae* Miq. are used as substitutes for *C. militaris* and *I. tenuipes*, respectively, because of their morphological similarities, including the color and shape of their fruiting bodies, and their host insect species. Thus, *C. militaris*, *C. pruinosa*, *I. tenuipes*, and *I. cicadae* are used to adulterate the herbal medicine Cordyceps (*O. sinensis*); *C. pruinosa* and *I. cicadae* are used as substitutes of *C. militaris* and *I. tenuipes*, respectively, in food products, and they are also used to adulterate the herbal medicine Cordyceps [3,6,7,8]. The various counterfeited products derived from these inauthentic species are consistently produced, and they adversely affect the safety, quality, and therapeutic efficacy of herbal medicines and dietary supplements [9].

Morphology-based identification of the authenticity and taxonomic origin of the source material in Cordyceps and related commercial products is usually impossible because the source material is typically used dried and powdered [9,10]. In addition, the relevant fungal species have distinct pharmacological properties, as summarized previously [1]. For example, *O. sinensis* is used for the tonification of the kidneys and lungs, to settle coughs and wheezing, and to inhibit sweating; on the other hand, *C. pruinosa* is used to treat stomach disorders, endotoxin shock, and inflammatory disease [1]. Therefore, reliable authentication tools for easy and quick identification of the taxonomic origins of Cordyceps and its related materials are consistently recommended in order to ascertain product safety, to regulate basic quality control, and as a counter-adulteration measure [6,11].

Diverse analytical methods are available for the quality standardization and identification of economically motivated adulterants in herbal and food materials. They are based on the assessment of taxonomic authenticity, including comparisons of morphological features, physiochemical properties, and DNA sequences [9]. Among these, DNA-based methods, such as DNA barcoding, random amplified polymorphic DNA, amplified fragment-length polymorphisms, and sequence characterized amplified region (SCAR) markers, are crucial, and these are widely used for the identification of the taxonomic origins of various herbal and food materials to the species level [12,13,14,15,16]. A DNA barcoding method based on universal DNA barcodes and a large sequence database plays a key role in the molecular discrimination of species, and the assessment of authenticity for herbal medicines and food supplements. This method is commonly used for sequence analysis of the internal transcribed spacer of the ribosomal DNA (nrDNA-ITS) in the fungal nuclear genome, the *mat*K and *rbc*L genes in the plant chloroplast genome, and the *COI* gene in the animal mitochondrial genome [12]. For over 10 years, numerous molecular genetic analyses involving nrDNA-ITS, the universal fungal DNA barcode, have been performed for the study of the phylogeny of Cordyceps and its related taxa, and for the identification of authentic Cordyceps [6]. However, these methods are limited to a simple and quick clarification of Cordyceps taxonomic origin, because the DNA barcoding method is complicated, and it involves techniques such as polymerase chain reaction (PCR) amplification, electrophoresis, gel isolation, sub-cloning (sometimes), sequencing, and database analysis. [6,17]. To overcome these limitations, a SCAR method combining DNA barcodes and SCAR marker development is attracting attention as an intuitive, simple, and reproducible molecular tool for determining the authenticity of the taxonomic origins of diverse herbal and food materials [9,18,19]. This SCAR method is widely used for the detection of substitutes and adulterants, and/or to assess the authenticity of precious herbal medicines and food supplements, such as *Panax*, saffron, and Cordyceps [6,20,21,22,23]. Lam et al. [6] used random amplified polymorphic DNA sequences to develop SCAR markers for a rapid identification of *O. sinensis*, and compared the specificity and experimental duration of this method with that of ITS DNA barcoding. However, the authors designed the SCAR marker only for *O. sinensis*, which does not address the issue of the market distribution of Cordyceps and related species in Korea. Real-time PCR is used in conjunction with SCAR markers in diverse fields for the identification of the taxonomic origins of pathogens, food ingredients and products, and herbal medicines, because it is fast, easy, and quantitative [24,25]. Real-time PCR is widely employed, and it is considered to be very useful for the detection of adulterants in food products and herbal medicines, such as pork, fish, ginseng, etc. [26,27,28,29].

Some genetic methods are currently available for distinguishing authentic Cordyceps species, specifically *O. sinensis*, from adulterants. Several *O. sinensis*-specific genetic markers that solely detect *O. sinensis* in host larvae and soil, were developed from the use of species-specific primers based on *O. sinensis* ITS sequences [6,30,31,32]. However, these *O. sinensis*-specific PCR assay methods could not identify the species of adulterants and contaminants. Liu et al. reported a set of species-specific primers that can identify six Cordyceps and related species, using *O. sinensis*, *C. militaris*, and *I. cicadae*, *C. gunnii*, *C. liangshanensis*, and *O. nutants* [3]. Although they developed species-specific markers for identifying the six species, including *O. sinensis*, *C. militaris*, and *I. cicadae*, the range of species that are distributed as inauthentic adulterants in the market are, however, different between Korea and China, and the detection limits of the adulterant species were not clarified [3]. Therefore, an additional marker is needed to discriminate between Cordyceps and its related species, including additional adulterant fungal species, and to improve the currently available genetic tools, in order for the markers to be used as simple and rapid assays of commercially processed products in Korea. In the current study, we analyzed the nrDNA-ITS sequences of Cordyceps, and its related medicinal and food fungal species, i.e., *O. sinensis*, *C. militaris*, *C. pruinosa*, *I. tenuipes*, and *I. cicadae*. We designed species-specific SCAR markers and used them for the development of conventional and real-time PCR assays. We also confirmed that these assays were able to successfully discriminate between each species in commercially processed products. The established assays will facilitate the quality control of Cordyceps and related products, providing a reliable tool for the prevention of product adulteration.

## 2. Results

### 2.1. Species Identification of Fungal Materials and Analysis of the nrDNA-ITS Sequences

To confirm the species identities and verify sequence variabilities, the complete nrDNA-ITS regions were amplified from 18 samples of six species (Table 1). DNA fragments were abundantly amplified from all templates and they had expected sizes of approximately 600 bp. The lengths of these PCR amplicons, determined after sub-cloning into the pGEM-T Easy vector, were 567 bp for *C. militaris*, 583 bp for *C. pruinosa*, 587 bp for both *I. cicadae* and *I. tenuipes*, 580 bp for *O. sinensis*, and 569 bp for *B. bassiana* (Table 2). Taxonomic origins of these 18 samples were confirmed by performing Basic Local Alignment Search Tool (BLAST) searches of the obtained sequences in both GenBank and BOLD databases. The data revealed that four of the fungal strains had been misidentified. Specifically, two presumed *I. tenuipes* samples, KACC43335 and KACC43338, were identified as being *I. cicadae* and *B. bassiana*, respectively, and two presumed *I. cicadae* samples, KACC44476 and KACC43334, were identified as being *I. tenuipes* and *B. bassiana*, respectively (Table 1). However, all other sample sequences matched their corresponding expected species, sharing over 99% sequence identity with their expected species. We also further confirmed the species of the fungal materials by an analysis of phylogenetic trees using 31 samples of nrDNA-ITS sequences obtained in the current study, and 13 that were retrieved from NCBI GenBank (Table 1, Figure S1, and Materials and Methods). A neighbor-joining analysis was used to classify the 31 nrDNA-ITS sequences into six distinct clades (*C. militaris*, *C. pruinosa*, *I. cicadae*, *I. tenuipes*, *O. sinensis*, and *B. bassiana*) and four genus cluster groups (*Cordyceps*, *Isaria*, *Ophiocordyceps*, and *Beauveria*) (Figure S1). These results strongly demonstrated that KACC13335, KACC44476, and KACC43334 and KACC43338 were *I. cicade*, *I. tenuipes*, and *B. bassiana*, respectively (Figure S1).

The nrDNA-ITS regions were compared using the multiple ClustalW tool in the BioEdit program [33]. The aligned sequences were 611 bp in length and had 56.06–62.24% GC content. Intraspecific sequence variability was only observed in two species, *C. militaris* and *O. sinensis*, at 0.0021 ± 0.0013 and 0.0023 ± 0.0020, respectively (Table 2). Interspecific sequence variability was between 0.0931 ± 0.0691 and 0.2136 ± 0.0095, with *I. cicadae* and *O. sinensis* harboring the most similar and the most diverged nrDNA-ITS sequences among the species, respectively (Table 2).

### 2.2. Development of the SCAR Markers and the Real-Time PCR Assays

**Figure 1:**
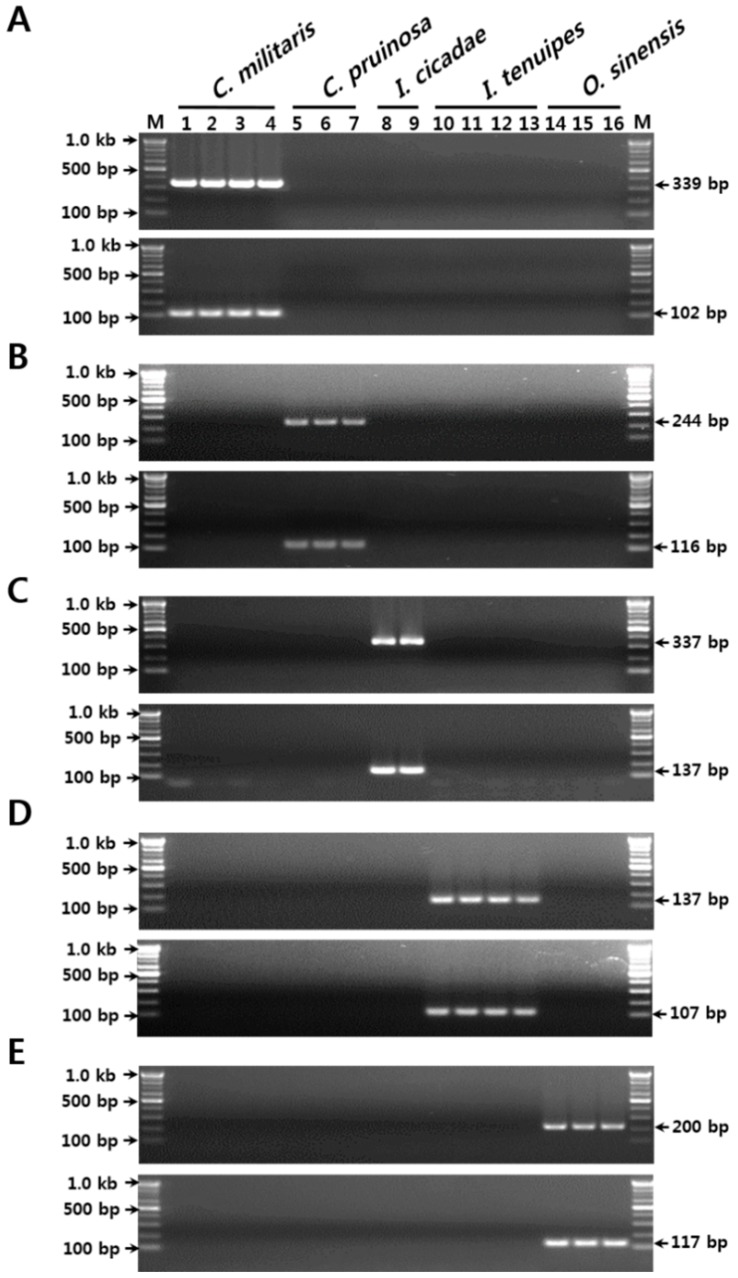
Development of the SCAR markers based on sequence variations in the nrDNA-ITS region. (**A**) Verification of primer specificities for *C. militaris*. (**B**) Verification of primer specificities for *C. pruinosa*. (**C**) Verification of primer specificities for *I. cicadae*. (**D**) Verification of primer specificities for *I. tenuipes*. (**E**) Verification of primer specificities for *O. sinensis*. The numbers 1–16 correspond to those listed in Table 1 in the “Gel lane” column. The precise lengths of the PCR products and DNA ladders are indicated to the right and left of the gel images, respectively. M, 100 bp DNA ladder.

To identify the optimal species-specific SCAR primers, the entire nrDNA-ITS sequence was analyzed, comparing the positions of nucleotide substitutions and indels between the species. Based on these nucleotide variabilities, we prepared several candidate SCAR primers and verified their specificities. The primer sets CM F2/CM R2 and CM F3/CM R3, designed to amplify 339-bp and 102-bp length *C. militaris*-specific amplicons, respectively, yielded DNA products only in the four samples that had been identified as *C. militaris*. No PCR products were obtained with the remaining 12 templates of the four related species, *C. pruinosa*, *I. cicadae*, *I. tenuipes*, and *O. sinensis* (Table 1 and Table 3, and Figure S2 and Figure 1A). These observations indicated that the two primer sets could be used to distinguish *C. militaris*, and medicinal and food materials containing *C. militaris*, from the four related species.

Two other primer sets, CP F2/CP R2 and CP F4/CP R3, yielded respective unique PCR amplicons of expected lengths (244 bp and 83 bp, respectively) from only the three *C. pruinosa* samples (Table 1 and Table 3, and Figure S2 and Figure 1B). Similarly, the two primer sets for each of the other three species (*I. cicadae*, *I. tenuipes*, and *O. sinensis*) yielded unique PCR products of expected sizes only from their respective target species, with no cross-reactivity with other species. These were the primer sets IC F1/IC R1 and IC F3/IC R3 for *I. cicadae* (337 bp and 139 bp amplicons, respectively), IT F4/IT R3, and IT F3/IT R2 for *I. tenuipes* (132 bp and 107 bp amplicons, respectively), and OS F1/OS R2 and OS F3/OS R3 for *O. sinensis* (200 bp and 117 bp amplicons, respectively) (Table 1 and Table 3, and Figure S2 and Figure 1C–E). These five sets of species-specific SCAR markers, each specific to two different target regions, can therefore be used to identify Cordyceps-related fungal species with high discriminability and stability, more efficiently and successfully than methods based on single SCAR markers.

**Figure 2:**
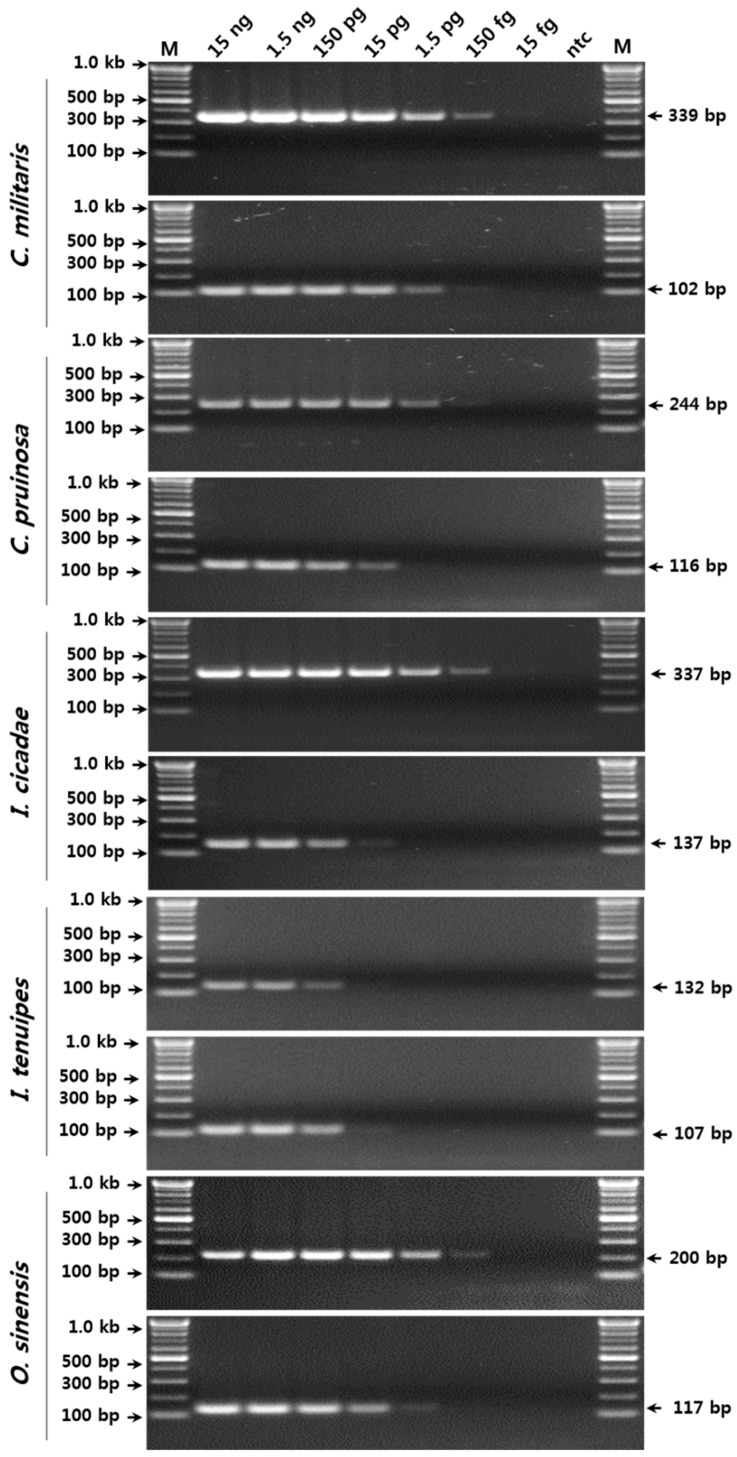
Verification of the detection limit of the SCAR markers using serial dilutions of template DNA. The precise lengths of the PCR products and the DNA ladders are indicated to the right and left of the gel images, respectively. M, 100 bp DNA ladder.

To verify the sensitivity and detection limits of the SCAR marker-based conventional PCR assay for the five species, serial 10-fold dilutions of pure genomic DNA (gDNA) (15 fg/μL to 15 ng/μL) were amplified using the respective SCAR primer sets (Figure 2). The amplification products with SCAR markers specific to *C. militaris*, *I. cicadae*, and *O. sinensis*, and to *I. tenuipes* were successfully obtained by conventional PCR in reactions containing ca. 150 fg and 15 pg of gDNA, respectively (Figure 2).

Standard curves of the species-specific SCAR markers and serial dilutions of the respective templates were used to establish a real-time PCR assay. To confirm the primer specificities for the real-time PCR assay, 15 ng of gDNA from the other four species were tested as non-target DNA; no cross-amplification was observed in any of the real-time PCR assays (Figure S3). The standard curves revealed high amplification efficiency and data linearity (Table 4 and Figure S3). The slopes of the standard curves were between −3.343 and −2.757, with the correlation coefficients being between 0.9587 and 0.9999 (Table 4). The Ct values were 20, 24, 28, 24, and 21 cycles for *C. militaris*, *C. pruinosa*, *I. cicadae*, *I. tenuipes*, and *O. sinensis*, respectively (Table 4). The sensitivities (LOD, limit of detection) of the real-time PCR assays were below 1.5 pg for all species (Figure S3).

### 2.3. Verification of the SCAR Markers and the Real-Time PCR Assay Using Commercial Products

**Figure 3:**
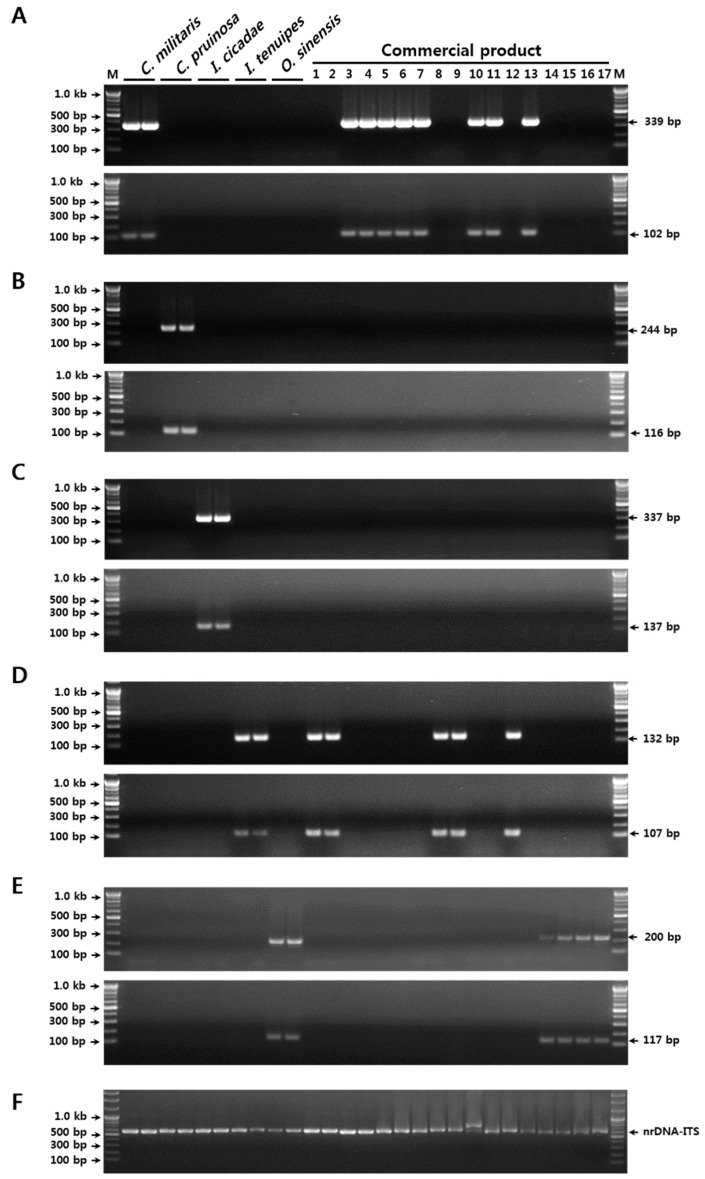
Validation of SCAR marker discriminability, and authentication of the taxonomic origin of resources in commercial Cordyceps and its related products. (**A**) Outcome of PCR amplification with the *C. militaris*-specific SCAR markers. (**B**) Outcome of PCR amplification with the *C. pruinosa*-specific SCAR markers. (**C**) Outcome of PCR amplification with the *I. cicadae*-specific SCAR markers. (**D**) Outcome of PCR amplification with the *I. tenuipes*-specific SCAR markers. (**E**) Outcome of PCR amplification with the *O. sinensis*-specific SCAR markers. (**F**) Outcome of PCR amplification with the nrDNA-ITS primers (ITS1 and ITS4) as control. Numbers 1–17 in the commercial products correspond to those listed in Table 5. The precise lengths of the PCR products and the DNA ladders are indicated to the right and left of the gel images, respectively. M, 100 bp DNA ladder.

Verification of the reproducibilities and discriminabilities of the SCAR markers developed in the current study was performed using 17 commercial herbal medicines and dietary supplements (Table 5). Thirteen samples of Cordyceps-related material, including seven dried herbal medicines, two fresh fruiting bodies, two dried powders, two mixed pill-type dietary supplements, and four dried Cordyceps herbal medicines, were purchased at markets in Korea, China, and Bhutan (Table 5). Of these samples, most products were labeled only by a common name, namely, Dong Chung Ha Cho or Cordyceps, without any specification of origin at the species level. Using the duplex SCAR markers and a real-time PCR assay, five out of the 11 samples that lacked species specification on the product label were authenticated as *C. militaris*-, two as *I. tenuipes*-, and four as *O. sinensis*-derived products (Table 5 and Figure 3). Further, one herbal medicine sample (voucher no. 2-2016-F020 in Table 5), that was labeled as a *Paecillomyces japonica* (a synonym of *I. japonica*) product, was also identified as *I. tenuipes* by both conventional and real-time PCR (Table 5 and Figure 3). For the other five products, the identities of the species specified on the product labels were confirmed (Table 5 and Figure 3). Interestingly, the original species in pill-type dietary supplements (Voucher nos. 2-2016-F024 and 2-2016-F024) that was presented as a mixture of diverse plant materials and cultured mycelium powder, were also identified as *I. tenuipes* and *C. militaris*, respectively (Table 5 and Figure 3). These observations indicated that the two sets of SCAR markers and the real-time PCR assay established in the current study could be used for the identification of the five Cordyceps and the related species, as the method was able to distinguish economically motivated adulterants from authentic Cordyceps in both commercially processed products and herbal medicines.

## 3. Discussion

The identification of authentic species has become a crucial issue for the quality control of food and medicinal sources, because most of these materials are collected from wild habitats or are cultivated on farms [34]. Since the accurate identification of fungal species is very difficult, depending on the conventional method used, more reliable and objective methods are required in order to discriminate between strains at the species level [3]. In this study, we developed a simple PCR assay method, and confirmed that the comparative analysis of nrDNA-ITS sequences is one of the most reliable tools for overcoming these difficulties. To verify the accurate determination of species in the samples listed in Table 1, we carried out BLAST searches, compared entire nrDNA-ITS sequences, and constructed phylogenetic trees. From the BLAST searches, we confirmed that four fungal samples had been misidentified (Table 1). We also further confirmed the species identification results by an analysis of sequences and phylogenetic trees (Figures S1 and S2). A phylogenetic tree of Cordyceps and five related fungal species was constructed from 31 nrDNA-ITS sequences, including 18 nrDNA-ITS sequences that were obtained in the current study and 13 retrieved from the NCBI GenBank, based on the neighbor-joining method (Table 1, and Materials and Methods). The neighbor-joining analysis classified the 31 nrDNA-ITS sequences into six distinct clades with over 70% bootstrap values (*C. militaris*, *C. pruinosa*, *I. cicadae*, *I. tenuipes*, *O. sinensis*, and *B. bassiana*), and four genus cluster groups (*Cordyceps*, *Isaria*, *Ophiocordyceps*, and *Beauveria*) (Figure S1). These observations strongly confirmed that the identification results were correctly verified, and that the nrDNA-ITS sequences could be used to distinguish Cordyceps and its five related taxa to the genus level, with the species showing a phylogenetic relationship similar to relationship reported earlier [35]. Further, the phylogenetic analysis confirmed that these six fungal species, which are used as important food and medicinal ingredients, may indeed be distinguished based on nrDNA-ITS sequence divergence.

During the development of species-specific SCAR markers, the specificity of the primer sequence is the most important, because nucleotide substitutions and/or indels play a crucial role for primer specificity [36]. To identify the optimal species-specific SCAR primers, the entire nrDNA-ITS sequences were analyzed, comparing the positions of nucleotide substitutions and indels between the species. While species-specific nucleotide substitutions and indels suitable for SCAR primer design were most numerous in the ITS1 and ITS2 regions of *C. militaris*, *C. pruinosa*, and *O. sinensis*, only several suitable positions were identified for *I. cicadae* and *I. tenuipes* (Figure S1). Therefore, both types of ITS regions were considered during the identification of candidate SCAR primers to distinguish Cordyceps and its related fungal species. Species-specific primers were designed based on these candidate regions, and their specificities for their respective target templates and species were verified using 16 samples, listed in Table 1. This led to the identification of two species-specific SCAR primer sets for each species, to increase the discriminability and stability of the molecular authentication method. Using these primers as a starting point, two SCAR markers were developed for each species, which yielded different sized PCR amplicons, with the expected sizes being obtained only during the analysis of the target species (Figure 1 and Table 3).

In addition, to verify the specificity of SCAR primers, the sequences of a total of 55 different species belonging to *Ophiocorcyceps* and closely related species—which were comprised of six species that were obtained in the current study, and 49 species that had been published in previous reports—were downloaded from GenBank, and the respective primer regions were compared with ClustalW (Figure S4) [3,30,31,32]. As a result, all primers had enough species-specific nucleotide substitutions in both the forward and reverse primer regions to provide primer specificity, with the exception of several species. The sequences of *C. roseostromata* in the CM F3 and CM R1 regions, and the sequences of *I. japonica* in the IT F4 and IT R3 also had one or two species-specific nucleotide substitutions. Thus, all SCAR primers that were developed in the current study included at least two species-specific nucleotide substitutions in one of the forward or reverse primer regions. These results also strongly supported the ability of the SCAR markers developed in this study to differentiate the five fungal species, and to discriminate between each precise species.

The detection of adulterants or contaminants is very important for the safety and quality control of food and medicinal materials [15]. In a previous report, an *O. sinensis*-specific SCAR marker was used to yield a PCR product from 8 ng of *O. sinensis* template gDNA in a conventional PCR assay; i.e., the sensitivity of that assay was inferior to the sensitivity of the current assay. However, the detection limits of the other species were not determined in order to check the quantity of adulteration and/or contamination [3]. In this study, we determined the detection limits for each species based on both conventional and real-time assays. Hence, the SCAR markers developed in the current study could be used to detect less than 0.1% (at least 0.0001%) contamination in a conventional PCR assay. Consequently, the assay might be employed for the purity assessment of herbal medicines and diverse food ingredients related to Cordyceps. These results indicated that a real-time PCR assay with SCAR markers was able to distinguish between the five fungal species with high sensitivity. Moreover, the assay may constitute a very efficient tool, not only for the identification of authentic *C. militaris*, *I. tenuipes*, and *O. sinensis* species and their closely related adulterants at the species level, but also for the detection of less than 0.01% contamination with adulterants or other species.

## 4. Materials and Methods

### 4.1. Fungal Material, DNA Extraction, and Sequencing

Thirteen fungal samples (four of *C. militaris*, three of *C. pruinosa*, two of *I. tenuipes*, and four of *I. cicadae*) were provided by the Korean Agricultural Culture Collection (KACC) and the Korean Collection for Type Cultures (KCTC), as listed in Table 1. *O. sinensis*, the Cordyceps herbal medicine, were provided by Prof. Gi-Ho Sung; they were collected in their native habitat in Bhutan (Table 1). Mycelia of the 13 fungal samples were obtained after growth on medium as suggested by the providers, consisting of 2% dextrose, 0.5% peptone, 0.5% yeast extract, and 1.5% agar. Fungal gDNA was extracted using the DNeasy plant mini kit (Qiagen, Valencia, CA, USA), gDNA from Cordyceps herbal medicines and commercial products was extracted from the fruiting bodies and individual components, respectively, using the same method, after processing the samples into a fine powder with a grinder (Precellys™ Grinder, Bertin Technologies, Montigny-le-Bretonneux, France). The concentration and purity of the extracted gDNA were determined using the NanoDrop ND-1000 spectrophotometer (NanoDrop, Wilmington, DE, USA), and by electrophoresis on 1.5% agarose gels with known standards. The final DNA concentrations in the samples were adjusted to approximately 15 ng/µL with TE buffer. The samples were stored at −20 °C for further analysis.

### 4.2. Phylogenetic Analysis

A phylogenetic tree was constructed using 31 complete nrDNA-ITS sequences in the MEGA7 program (Version 7.0.26). In addition to the 18 sequences obtained in the current study, 13 previously reported nrDNA-ITS sequences were retrieved from the GenBank, as follows: three *I. tenuipes* sequences (AB086215, AB086224, and EF411223) and two sequences each from *C. pruinosa* (AJ039338 and AY491995), *C. militaris* (AB084156 and AB255603), *I. cicadae* (KX017277 and KP771871), *Beauveria bassiana* (KU702657 and GU233698), and *O. sinensis* (EU570943 and AB067713). A phylogenetic tree was constructed using the neighbor-joining method and Kimura’s two-parameter model, with pairwise deletion for gaps and/or missing data, and 1000 replications for bootstrapping. *Nectria cinnabarina* (AB237663) was used as an outgroup control [35,37].

### 4.3. PCR Amplification of nrDNA-ITS and Species Identification

The nrDNA-ITS regions, including the 5.8S rRNA gene, were amplified in a 50 μL PCR mixture containing approximately 15 ng of gDNA, 0.4 µM each of the primers ITS1 and ITS4, and Solg™ 2 × Taq PCR Smart Premix 1 (Solgent, Daejeon, Korea), using a Pro Flex PCR system (Applied Biosystems, Waltham, MA, USA) as previously described [38]. The PCR products were separated on a 1.5% agarose gel. The target amplicons were then isolated using a gel extraction kit (Qiagen, Valencia, CA, USA) and sub-cloned into the pGEM™-T Easy vector system (Promega, Madison, WI, USA) following the manufacturer’s instructions. The inserted DNA fragments were sequenced using the primers SP6 and T7, using an ABI3730 DNA sequence analyzer (Applied Biosystems, Waltham, MA, USA). To check for the presence of PCR errors and misreads, five inserted PCR products were analyzed for each sample, and the sequences were manually edited after alignment, as previously described [17]. The identities of each species in the individual samples were confirmed using a BLAST-based comparison and similarity analysis involving the obtained sequences, and sequences were deposited in the NCBI GenBank and BOLD databases (Table 1). Species identity was further confirmed by a comparison of nucleotide sequences at the intra- and inter-species levels using the entire suite of nrDNA-ITS sequences listed in Table 1.

### 4.4. Analysis of the nrDNA-ITS Sequences and Development of SCAR Markers

To identify the species-specific nucleotide variants in the nrDNA-ITS region that could be used for SCAR marker development, 18 sample sequences representing six species, including *B. bassiana* (Bals.-Criv.) Vuill., were aligned and manually edited using the ClustalW algorithm in the BioEdit program (Version 7.2.5). The inter- and intraspecific variabilities were then analyzed using the MEGA7 program [33,37]. The resulting species-specific regions with distinct indels and nucleotide substitutions were selected as potential candidate SCAR primers, and they were synthesized to amplify the SCAR regions. A PCR was performed to confirm the specificities of individual primer sets, in 20 μL reaction mixtures containing approximately 15 ng of gDNA, and 0.4 µM of each species-specific forward and reverse primers. The amplification reactions were performed using the Pro Flex PCR system (Applied Biosystems, Waltham, MA, USA). The amplification conditions were as follows: initial denaturation at 95 °C for 2 min, followed by 35 cycles of 95 °C for 30 s, 63 °C for 30 s, and 72 °C for 30 s, and a final extension step at 72 °C for 5 min. To verify the PCR results, PCR products were resolved by 1.5% agarose gel electrophoresis, and the specificity and size of the DNA fragments were verified with a 100 bp DNA ladder (Solgent, Daejeon, Korea). To improve the stability and discriminability of the SCAR marker-based species identification method, two sets of SCAR primers were developed for each species, and the specificities of both SCAR markers were confirmed using the 18 samples listed in Table 1.

### 4.5. Establishment of a SYBR Green Real-Time PCR Assay

Real-time PCR was performed using a Qiagen Rotor-Gene Q thermal cycler (Qiagen, Valencia, CA, USA) with SYBR green, in 20-μL reaction volumes. To generate a standard curve for each species, real-time PCR reactions were performed in 20 μL reaction mixtures containing 1 × QuantiNova™ SYBR green PCR master mix, templates (one of the following five 10-fold serial dilutions for each species: 15 ng, 1.5 ng, 150 pg, 15 pg, or 1.5 pg), and 0.7 µM of each of the following primer pairs: CM F3/R3 for *C. militaris*, CP F4/R3 for *C. pruinosa*, IC F3/R2 for *I. cicadae*, IT F3/R2 for *I. tenuipes*, and OS F3/R3 for *O. sinensis*. gDNAs extracted from pure fungal cultures of each species were used as templates. Negative control reactions were performed in the absence of template DNA. The real-time PCR amplification conditions were as follows: a pre-denaturation step at 95 °C for 2 min; followed by 40 cycles of 95 °C for 10 s, and 55 °C for 20 s. The amplification program finished with a melting curve analysis, i.e., a progressive denaturation of PCR products from 60 °C to 99 °C, at a rate of 1 °C every 5 s. The efficiency (E) of the real-time PCR amplification was calculated from the slope of the standard curve using the equation E = (10 − 1/slope − 1) × 100. The threshold cycle (Ct) was calculated from the slope of the standard curve for each species, using Rotor-Gene Q series software (Version 2.1; Qiagen).

### 4.6. Verification of the SCAR Markers and Real-Time PCR Assay Using Commercial Products

To validate the SCAR markers and the real-time PCR assay, 17 commercial Cordyceps and related products were purchased from markets in Korea, Bhutan, and China, and their taxonomic origins were verified. About 15 ng of total gDNA extracted from individual samples was used as a template in 20-μL reaction mixtures. The SCAR PCR amplification was performed under the same conditions as described for developing the SCAR markers, with each of the 0.5 µM primers that are listed in Table 3. As a PCR amplification control, we also amplified nrDNA-ITS regions for all of the 17 commercial products using ITS1 and ITS4 primers. The specificities of the SCAR markers and the taxonomic origins of the 17 commercial samples were confirmed by 1.5% agarose gel electrophoresis, depending on the amplicon size and specificity, and they were reconfirmed by comparing the resulting sequences of the PCR products (SCAR and nrDNA-ITS products) with the control nrDNA-ITS sequences after gel isolation and sub-cloning into the pGEM-T Easy vector (Promega, Madison, WI, USA). BLAST analyses of the SCAR and nrDNA-ITS PCR product sequences were also conducted to confirm the specificities of the PCRs and the identity of the respective species, using BLAST-based comparison and similarity analysis involving the obtained sequences, as well as sequences deposited in the NCBI GenBank and BOLD databases. Real-time amplification was performed as described above, with approximately 15 ng of template DNA, extracted from each of the 17 commercial samples.

### 4.7. Data Availability

The finalized representative nrDNA-ITS sequences obtained from the 18 samples representing the five fungi species (Cordyceps and its related species) were deposited in the NCBI GenBank database under the following accession numbers: *O. sinensis*, MG833285–MG833287; *C. militaris*, MG833281–MG833284; *C. pruinosa*, MG833288–MG833290; *I. tenuipes*, MG833293–MG833296; *I. cicadae*, MG833291–MG833292; and *B. bassiana*, MG833297–MG833298.

## 5. Conclusions

In the current study, we obtained the nrDNA-ITS sequences of five Cordyceps and related medicinal fungal species, *O. sinensis*, *C. militaris*, *C. pruinosa*, *I. tenuipes*, and *I. cicadae*. Based on these sequences, we developed two sets of SCAR markers for identifying each species and for detecting the contamination of inauthentic adulterants. Using these species-specific SCAR markers, we also established a real-time PCR assay that is capable of identifying the taxonomic origin, the degree of contamination (purity), and the amount of the adulterants in samples. The SCAR-based conventional and real-time PCR assays were also verified using commercially processed food products and herbal medicines. These assays may be used to identify authentic fungal species and to prevent the agricultural, industrial, and therapeutic applications of inauthentic *O. sinenesis.* In addition, it may constitute a reliable tool for the quality control and safety assurance of medicinal and food ingredients for Cordyceps and its related materials.

## Figures and Tables

**Table 1:** Fungal material used in the current study.

Source	Name		Species Identification	Location	GenBank Number	Gel Lane
Registered No.	Registered Name	Current Name				
KACC 43316	*C. militaris*	*C. militaris*	*C. militaris*	Jeju, Korea	MG833296	1
KACC 43319				Youngju, Korea	MG833296	2
KACC 44463				Cheongyang, Korea	MG833296	3
KCTC 6064				Unknown, Korea	MG833296	4
KACC 43329	*C. pruinosa*	*C. pruinosa*	*C. pruinosa*	Cheongyang, Korea	MG833296	5
KACC 43333				Hoengseong, Korea	MG833296	6
KACC 43331				Jecheon, Korea	MG833296	7
KACC 43335	*P. tenuipes*	*I. tenuipes*	*I. cicadae*	Yanju, Korea	MG833296	8
KACC 44107	*P. cicadae*	*I. cicadae*		Jeju, Korea	MG833296	9
KACC 43336	*P. tenuipes*	*I. tenuipes*	*I. tenuipes*	Hoengseong, Korea	MG833296	10
KACC 51995				Suwon, Korea	MG833296	11
KACC 52194				Jeju, Korea	MG833296	12
KACC 44476	*P. cicadae*	*I. cicadae*		Inje, Korea	MG833296	13
2-2016-F027	*C. sinensis*	*O. sinensis*	*O. sinensis*	Paro, Bhutan	MG833296	14
2-2016-F028				Thimphu, Bhutan	MG833296	15
2-2016-F029				Unknown, China	MG833296	16
KACC 43338	*P. tenuipes*	*I. tenuipes*	*B. bassiana*	Pyeongchang, Korea	MG833296	17
KACC 43334	*P. cicadae*	*I. cicadae*		Jangseong, Korea	MG833296	18

**Table 2:** Characteristics of the internal transcribed spacer of the ribosomal DNA (nrDNA-ITS) sequences.

Species	Constant Length (bp)	Aligned Length (bp)	Intraspecific Variability	Interspecific Variability	GC Content (%)
*C. militaris*	567	611	0.0021 ± 0.0013	0.1136 ± 0.0534	56.57
*C. pruinosa*	583	611	0.0000 ± 0.0000	0.1062 ± 0.0462	57.98
*I. cicadae*	587	611	0.0000 ± 0.0000	0.0931 ± 0.0691	59.11
*I. tenuipes*	587	611	0.0000 ± 0.0000	0.1093 ± 0.0629	59.97
*O. sinensis*	580	611	0.0023 ± 0.0020	0.2136 ± 0.0095	62.24
*B. bassiana*	569	611	0.0000 ± 0.0000	0.1028 ± 0.0579	56.06

**Table 3:** Sequences of the sequence characterized amplified region (SCAR) primers, and the sizes and specificity of the amplified DNA fragments.

Primer Name	Primer Sequence (5′→3′)	Amplicon Size (bp)	Species Specificity
CM F2	GGCCCCAAACAGTGTATCTAC	339	*C. militaris*
CM R2	CCGGTGCGAGTTGGCGTACTA		
CM F3 *	CAACCCTTTGTGAACATACCT	102	
CM R3 *	GTAGATACACTGTTTGGGGCC		
CP F2	GACCCCAAACTCTGTTTCTAG	244	*C. pruinosa*
CP R2	CCCCGCGAGGAGGGGTCGAGT		
CP F1 *	ACTCGACCCCTCCTCGCGGGG	116	
CP R1 *	GTCCCGGTGCGACTGGTGTG		
IC F1	ACGCAACCCTGTATCCATCAG T	337	*I. cicadae*
IC R1	TTCCCGGTGCGACTGGTTGT		
IC F3 *	ACCCTTCTGTGAACCTACGCATC	137	
IC R3 *	GATTCAGCGAGACTGATGGAT		
IT F4 *	CCTTCTGTGAACCTACCCATA	132	*I. tenuipes*
IT R3 *	GAGCGGCTCACAGATACAGG		
IT F3	CCATACTTGCTTCGGCGGACC	107	
IT R2	GCTCACAGATACAGGGTTGC		
OS F1 *	AGCGTCATCTCAACCCTCGAG	200	*O. sinensis*
OS R2 *	TGATCCGAGGTCAACTGGAGG		
OS F3	GAACACCACAGCAGTTGCCT	117	
OS R3	GCTTCTTGACTGAGAGATGCC		

Asterisks (*) indicate SCAR markers used in the development of the real-time polymerase chain reaction (PCR) assay.

**Table 4:** The sensitivities, efficiencies, and correlation coefficients of the real-time PCR assays.

Sample	Ct Value					Efficiency	R^2^	Slope
	15 ng	1.5 ng	150 pg	15 pg	1.5 pg			
*C. militaris*	7.55	10.30	13.65	17.27	20.75	99	0.99759	−3.337
*C. pruinosa*	11.27	14.02	17.60	21.28	24.46	98	0.99783	−3.364
*I. cicadae*	14.72	17.97	21.47	24.76	28.19	98	0.99990	−3.372
*I. tenuipes*	13.89	14.62	17.50	20.88	24.55	131	0.95865	−2.757
*O. sinensis*	8.31	11.66	15.06	18.68	21.98	96	0.99981	−3.434

**Table 5:** The sensitivities, efficiencies, and correlation coefficients of the real-time PCR assays.

No.	Voucher No.	Product Name (Species)	Product Form	Identification	Quantity (ng/μL)	Country
1	2-2016-F013	DCHC (not specified) **	Dried food ingredient	*I. tenuipes*	29.8	Korea
2	2-2016-F014	Yellow DCHC (not specified) **	Dried food ingredient	*I. tenuipes*	10.8	Korea
3	2-2016-F015	Red DCHC (not specified) **	Dried herbal medicine	*C. militaris*	11.4	Korea
4	2-2016-F016	DCHC (not specified) **	Dried herbal medicine	*C. militaris*	2.2	Korea
5	2-2016-F017	DCHC Cho (not specified) **	Fresh fruiting body	*C. militaris*	6.3	Korea
6	2-2016-F018	DCHC (not specified) **	Fresh fruiting body	*C. militaris*	4.7	Korea
7	2-2016-F019	DCHC (*C. militaris*) *	Dried herbal medicine	*C. militaris*	1.9	Korea
8	2-2016-F020	DCHC (*P. japonica*) *	Dried food ingredient	*I. tenuipes*	7.2	Korea
9	2-2016-F021	DCHC (*I. tenuipes*) *	Dried herbal medicine	*I. tenuipes*	11.2	Korea
10	2-2016-F022	DCHC (not specified) **	Dietary supplement (dried powder)	*C. militaris*	0.2	Korea
11	2-2016-F023	DCHC (*C. militaris*) *	Dietary supplement (dried powder)	*C. militaris*	2.1	Korea
12	2-2016-F024	DCHC (*I. tenuipes*) *	Dietary supplement (mixed pill)	*I. tenuipes*	8.2	Korea
13	2-2016-F025	DCHC (*C. militaris*) *	Dietary supplement (mixed pill)	*C. militaris*	0.9	Korea
14	2-2016-F026	DCHC (not specified) **	Dried herbal medicine	*O. sinensis*	3.4	Bhutan
15	2-2016-F032	DCHC (not specified) **	Dried herbal medicine	*O. sinensis*	6.7	China
16	2-2016-F033	DCHC (not specified) **	Dried herbal medicine	*O. sinensis*	1.6	China
17	2-2016-F034	DCHC (not specified) **	Dried herbal medicine	*O. sinensis*	4.5	China

DCHC indicates an abbreviation of *Dong Chung Ha Cho*. * Species listed on the product label. ** The taxonomic origin was not specified on the product label.

## Supplementary Materials

The following are available online. Figure S1, Confirmation of species identification for six fungal species by the construction of a phylogenetic tree based on their nrDNA-ITS sequences; Figure S2, Comparison of nrDNA-ITS sequences and positions of species-specific primers for the development of SCAR markers; Figure S3, Establishment of a real-time PCR assay for distinguishing Cordyceps and its related species, and the results from real-time PCR assays of commercial products; Figure S4, Confirmation of primer specificity by the alignment of species-specific SCAR primer regions used for both conventional and real-time PCR assays.

## References

[1] Kuo H.C., Su T.L., Yang H.L., Chen T.Y. (2005). Identification of chinese medicinal fungus *Cordyceps sinensis* by PCR single stranded confromation polymorphism and phylogenetic relationship. J. Agric. Food Chem..

[2] Korea Institute of Oriental Medicine Defining Dictionary for Medicinal Herbs. http://boncho.kiom.re.kr/codex/.

[3] Liu Y., Wang X.Y., Gao Z.T., Han J.P., Xiang L. (2017). Detection of *Ophiocordyceps sinensis* and Its Common Adulterates Using Species-Specific Primers. Front. Microbiol..

[4] Korea Food & Drug Administration Korean Food Standards Codex (Food Material). https://www.foodsafetykorea.go.kr/portal/safefoodlife/foodMeterial/foodMeterialDB.do?menu_grp=MENU_NEW04&menu_no=2968.

[5] Zhou X., Gong Z., Su Y., Lin J., Tang K. (2009). Cordyceps fungi: Natural products, pharmacological functions and developmental products. J. Pharm. Pharmacol..

[6] Lam K.Y., Chan G.K., Xin G.Z., Xu H., Ku C.F., Chen J.P., Yao P., Lin H.Q., Dong T.T., Tsim K.W. (2015). Authentication of *Cordyceps sinensis* by DNA Analyses: Comparison of ITS Sequence Analysis and RAPD-Derived Molecular Markers. Molecules.

[7] Inglis P.W., Myrian S.T. (2006). Identification and taxonomy of some entomopathogenic *Paecilomyces* spp.(Ascomycota) isolates using rDNA-ITS sequences. Genet. Mol. Biol..

[8] Li S.P., Yang F.Q., Tsim K.W. (2006). Quality control of *Cordyceps sinensis*, a valued traditional Chinese medicine. J. Pharm. Biomed. Anal..

[9] Hong E., Lee S.Y., Jeong J.Y., Park J.M., Kim B.H., Kwon K., Chun H.S. (2017). Modern Analytical Methods for the Detection of Food Fraud and Adulteration by Food Category. J. Sci. Food Agric..

[10] Daria S., Rosa R. (2014). DNA Markers for Food Products Authentication. Diversity.

[11] Xiang L., Song J., Xin T., Zhu Y., Shi L., Xu X., Pang X., Yao H., Li W., Chen S. (2013). DNA barcoding the commercial Chinese caterpillar fungus. FEMS Microbiol. Lett..

[12] Chen S., Pang X., Song J., Shi L., Yao H., Han J., Leon C. (2014). A renaissance in herbal medicine identification: From morphology to DNA. Biotechnol. Adv..

[13] Sheorey R.R., Tiwati A. (2011). Random amplified polymorphic DNA (RAPD) for identification of herbal materials and medicine—A review. J. Sci. Ind. Res..

[14] Vos P., Hogers R., Bleeker M., Reijans M., van de Lee T., Hornes M., Friters A., Pot J., Paleman J., Kuiper M. (1995). AFLP: A new technique for DNA fingerprinting. Nucleic Acids Res..

[15] Kim W.J., Moon B.C., Yang S., Han K.S., Choi G., Lee A.Y. (2016). Rapid Authentication of the Herbal Medicine Plant Species *Aralia continentalis* Kitag. and *Angelica biserrata* CQ Yuan and RH Shan Using ITS2 Sequences and Multiplex-SCAR Markers. Molecules.

[16] Lee Y.M., Ji Y., Kang Y.M., Kim W.J., Choi G., Moon B.C. (2016). Molecular authentication of Pinelliae Tuber and its common adulterants using RAPD-derived multiplex sequence characterized amplified region (multiplex-SCAR) markers. Int. J. Clin. Exp. Med..

[17] Moon B.C., Kim W.J., Han K.S., Yang S., Kang Y.M., Park I., Piao R. (2017). Differentiating Authentic Adenophorae Radix from Its Adulterants in Commercially-Processed Samples Using Multiplexed ITS Sequence-Based SCAR Markers. Appl. Sci..

[18] Heubl G. (2010). New aspects of DNA-based authentication of Chinese medicinal plants by molecular biological techniques. Planta Med..

[19] Bhagyawant S.S. (2016). RAPD-SCAR Markers: An Interface Tool for Authentication of Traits. J. Biosci. Med..

[20] Choi Y.E., Ahn C.H., Kim B.B., Yoon E.S. (2008). Development of species specific AFLP-derived SCAR marker for authentication of *Panax japonicus* C. A. MEYER. Biol. Pharm. Bull..

[21] Torelli A., Marieschi M., Bruni R. (2014). Authentication of saffron (*Crocus sativus* L.) in different processed, retail products by means of SCAR markers. Food Control.

[22] Wolff K., Schoen E.D., Rijn J.P. (1993). Optimizing the generation of random amplified polymorphic DNAs in chrysanthemum. Theor. Appl. Genet..

[23] Babaei S., Talebi M., Bahar M. (2014). Developing an SCAR and ITS reliable multiplex PCR-based assay for safflower adulterant detection in saffron samples. Food Control.

[24] Al-Kahtani H.A., Ismail E.A., Ahmed M.A. (2017). Pork detection in binary meat mixtures and some commercial food products using conventional and real-time PCR techniques. Food Chem..

[25] Gao L., Yu H.X., Kang X.H., Shen H.M., Li C., Liu T.G., Liu B., Chen W.Q. (2016). Development of SCAR Markers and an SYBR Green Assay to Detect *Puccinia striiformis* f. sp. *tritici* in Infected Wheat Leaves. Plant Dis..

[26] Taboada L., Sanchez A., Sotelo C.G. (2017). A new real-time PCR method for rapid and specific detection of ling (*Molva molva*). Food Chem..

[27] Cammà C., Di Domenico M., Monaco F. (2012). Development and validation of fast Real-Time PCR assays for species identification in raw and cooked meat mixtures. Food Control.

[28] Mano J., Nishitsuji Y., Kikuchi Y., Fukudome S.I., Hayashida T., Kawakami H., Kurimoto Y., Noguchi A., Kondo K., Teshima R. (2017). Quantification of DNA fragmentation in processed foods using real-time PCR. Food Chem..

[29] Jung J., Kim K.H., Yang K., Bang K.H., Yang T.J. (2014). Practical application of DNA markers for high-throughput authentication of *Panax ginseng* and *Panax quinquefolius* from commercial ginseng products. J. Ginseng Res..

[30] Jin G.S., Wang X.L., Li Y., Wang W.J., Yang R.H., Ren S.Y., Yao Y.J. (2013). Development of conventional and nested PCR assays for the detection of *Ophiocordyceps sinensis*. J. Basic Microbiol..

[31] Peng Q., Zhong X., Lei W., Zhang G., Liu X. (2013). Detection of *Ophiocordyceps sinensis* in soil by quantitative real-time PCR. Can. J. Microbiol..

[32] Lei W., Li S., Peng Q., Zhang G., Liu X. (2013). A real-time qPCR assay to quantify *Ophiocordyceps sinensis* biomass in Thitarodes larvae. J. Microbiol..

[33] Hall T.A. (1999). BioEdit: A User-Friendly Biological Sequence Alignment Editor and Analysis Program for Windows 95/98/NT. Nucleic Acids Symposium Series.

[34] Ganie S.H., Upadhyay P., Das S., Sharma M.P. (2015). Authentication of medicinal plants by DNA markers. Plant Gene.

[35] Sung G.H., Hywel-Jones N.L., Sung J.M., Luangsa-Ard J.J., Shrestha B., Spatafora J.W. (2007). Phylogenetic classification of Cordyceps and the clavicipitaceous fungi. Stud. Mycol..

[36] Semagn K., Bjørnstad Å., Ndjiondjop M. (2006). An overview of molecular marker methods for plants. Afr. J. Biotechnol..

[37] Kumar S., Stecher G., Tamura K. (2016). MEGA7: Molecular Evolutionary Genetics Analysis Version 7.0 for Bigger Datasets. Mol. Biol. Evol..

[38] White T.J., Bruns T., Lee S., Taylor J. (1990). PCR Protocols: A Guide to Methods and Applications.

