# Supplementary material for: Establishment of a PCR Assay for the Detection and Discrimination of Authentic Cordyceps and Adulterant Species in Food and Herbal Medicines
Source: Molecules. 2018 Aug 2;23(8):1932. doi: 10.3390/molecules23081932 (PMC6222412; doi:10.3390/molecules23081932)
Supplement: Supplementary file 1 [file molecules-23-01932-s001.pdf]

molecules

Establishment of a PCR Assay for the Detection and Discrimination of Authentic Cordyceps and Adulterant Species in Food and Herbal Medicines

Byeong Cheol Moon [1,*], Wook Jin Kim [1], Inkyu Park [1], Gi-Ho Sung [2,3] and Pureum Noh [1]

* Correspondence: bcmoon@kiom.re.kr; Tel.: +82-42-868-9530

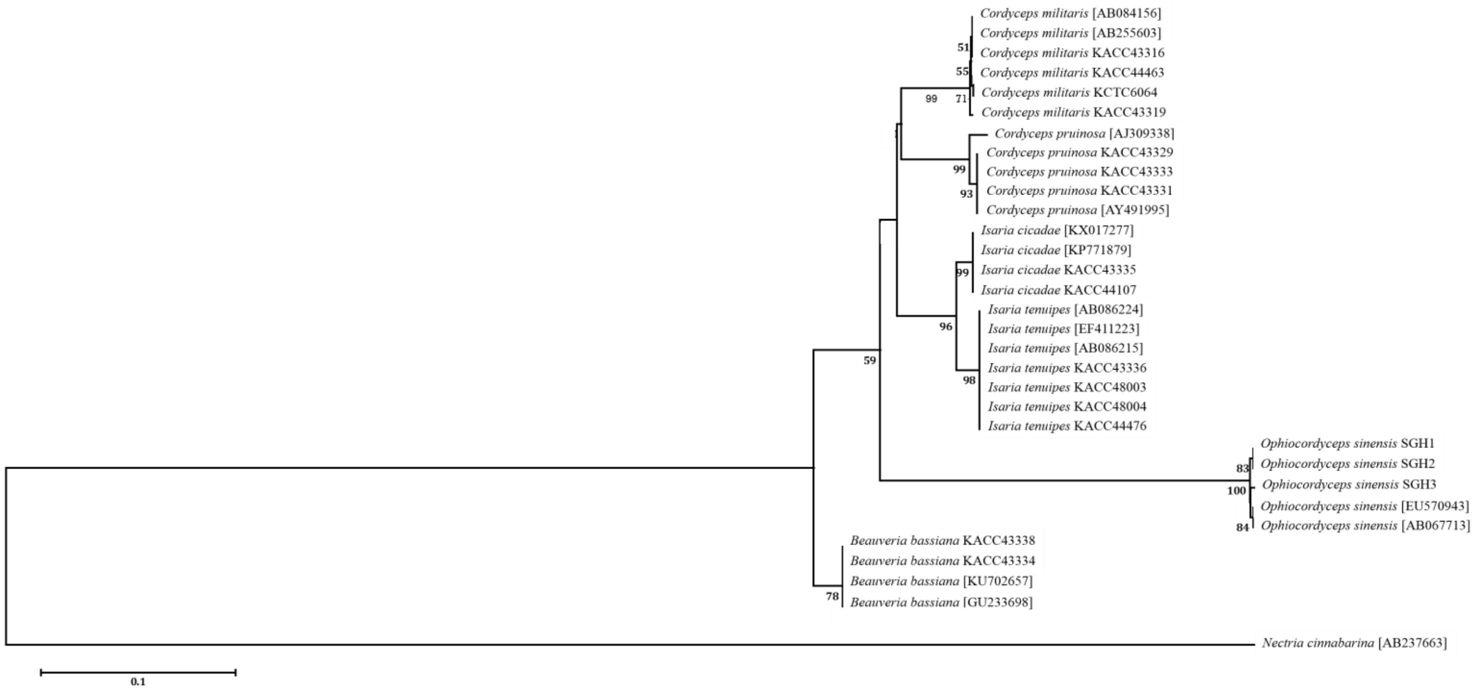

Figure S1. Phylogenetic relationships between six fungal species (Cordyceps and related species, and a closely related medicinal fungus) based on their nrDNA-ITS sequences. Numbers at the nodes are the bootstrap values from 1000 replicates. The nrDNA-ITS sequence of *Nectria cinnabarina* (AB237663) was used as an outgroup.

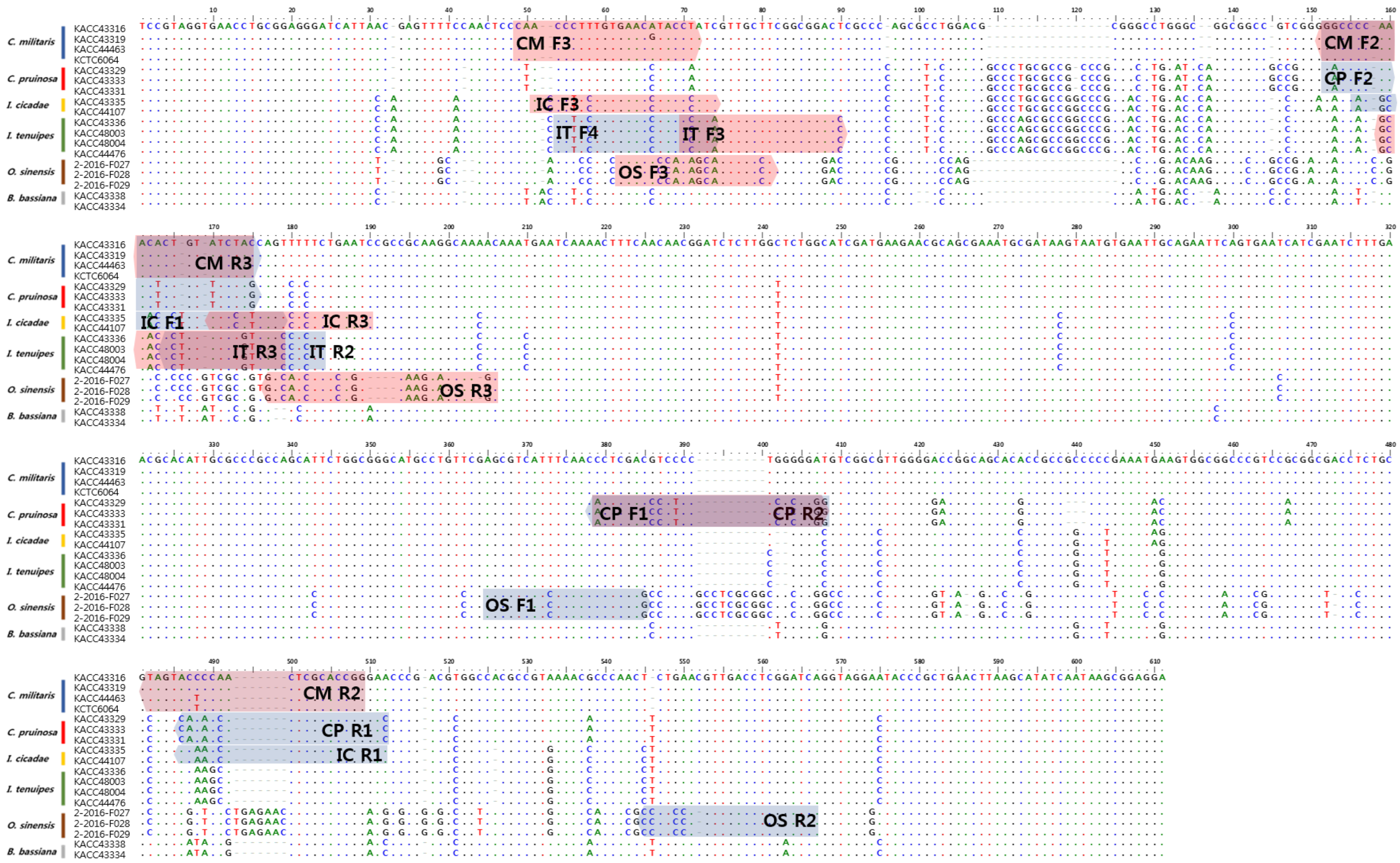

Figure S2. Comparison of nrDNA-ITS sequences and positions of species-specific primers for the development of SCAR markers. Sequences were aligned using the ClustalW alignment tool of the BioEdit program (version 7.2.6). The arrow boxes indicate primer sequences and their polarities. The primer names are given in the boxes. Dots (.) and dashes (-) indicate identical nucleotides and gaps, respectively, when compared with C. militaris KACC 43316, to maximize sequence alignment presentation.

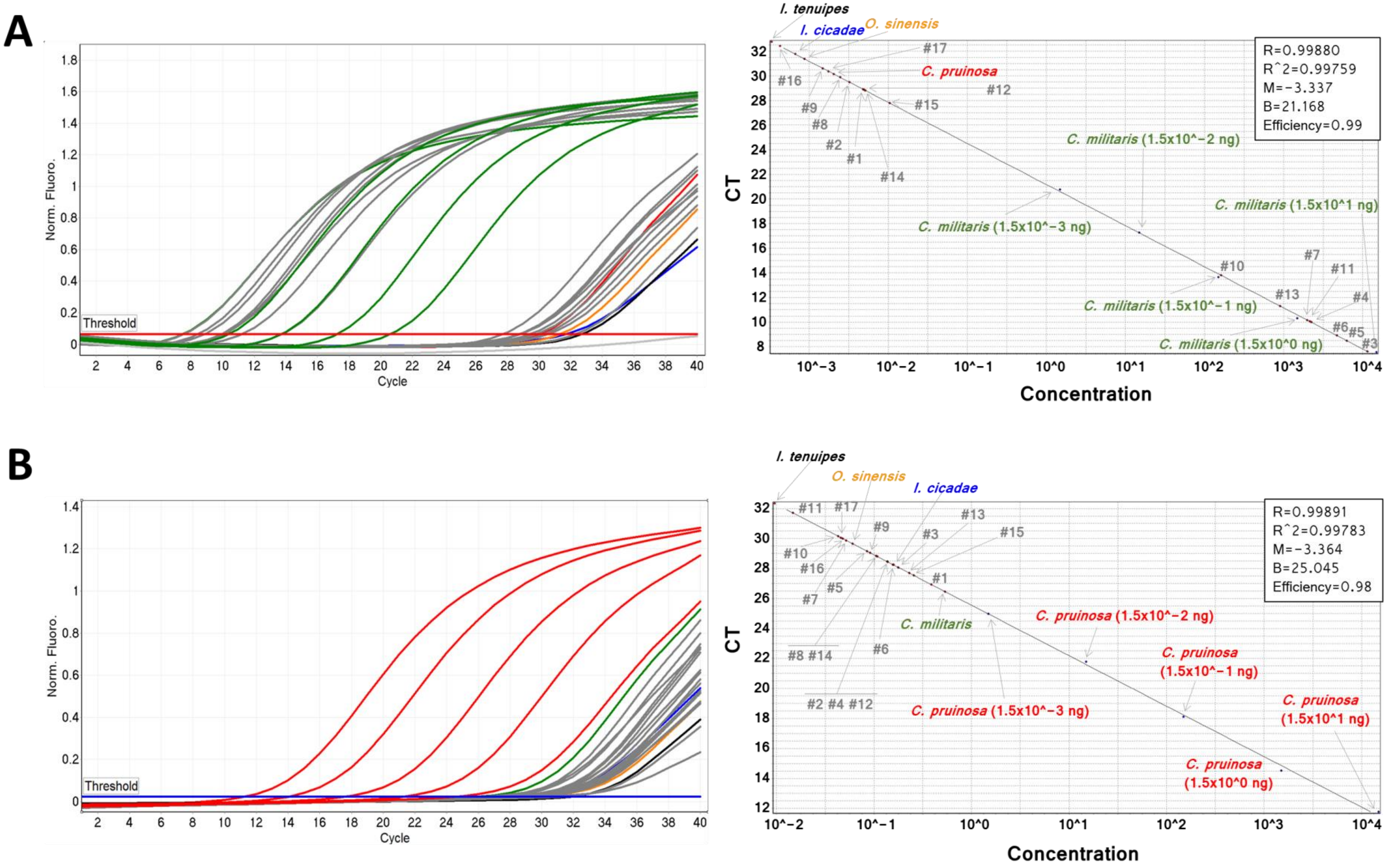
A
Norm. Fluoro.
Threshold
Cycle
I. tenuipes
I. cicadae
O. sinensis
C. pruinosa
#17
#16
#12
#9
#8
#15
#2
#1
#14
R=0.99880
R^2=0.99759
M=−3.337
B=21.168
Efficiency=0.99
C. militaris (1.5x10^−2 ng)
C. militaris (1.5x10^1 ng)
C. militaris (1.5x10^−3 ng)
#10
#7
#11
#13
#4
C. militaris (1.5x10^−1 ng)
#6
#5
C. militaris (1.5x10^0 ng)
#3
CT
Concentration
B
Norm. Fluoro.
Threshold
Cycle
I. tenuipes
O. sinensis
I. cicadae
#11
#17
#9
#13
#3
#15
#10
#16
#5
#7
#1
C. militaris
C. pruinosa (1.5x10^−2 ng)
#8 #14
#6
C. pruinosa (1.5x10^−1 ng)
#2 #4 #12
C. pruinosa (1.5x10^−3 ng)
C. pruinosa (1.5x10^1 ng)
C. pruinosa (1.5x10^0 ng)
R=0.99891
R^2=0.99783
M=−3.364
B=25.045
Efficiency=0.98
CT
Concentration

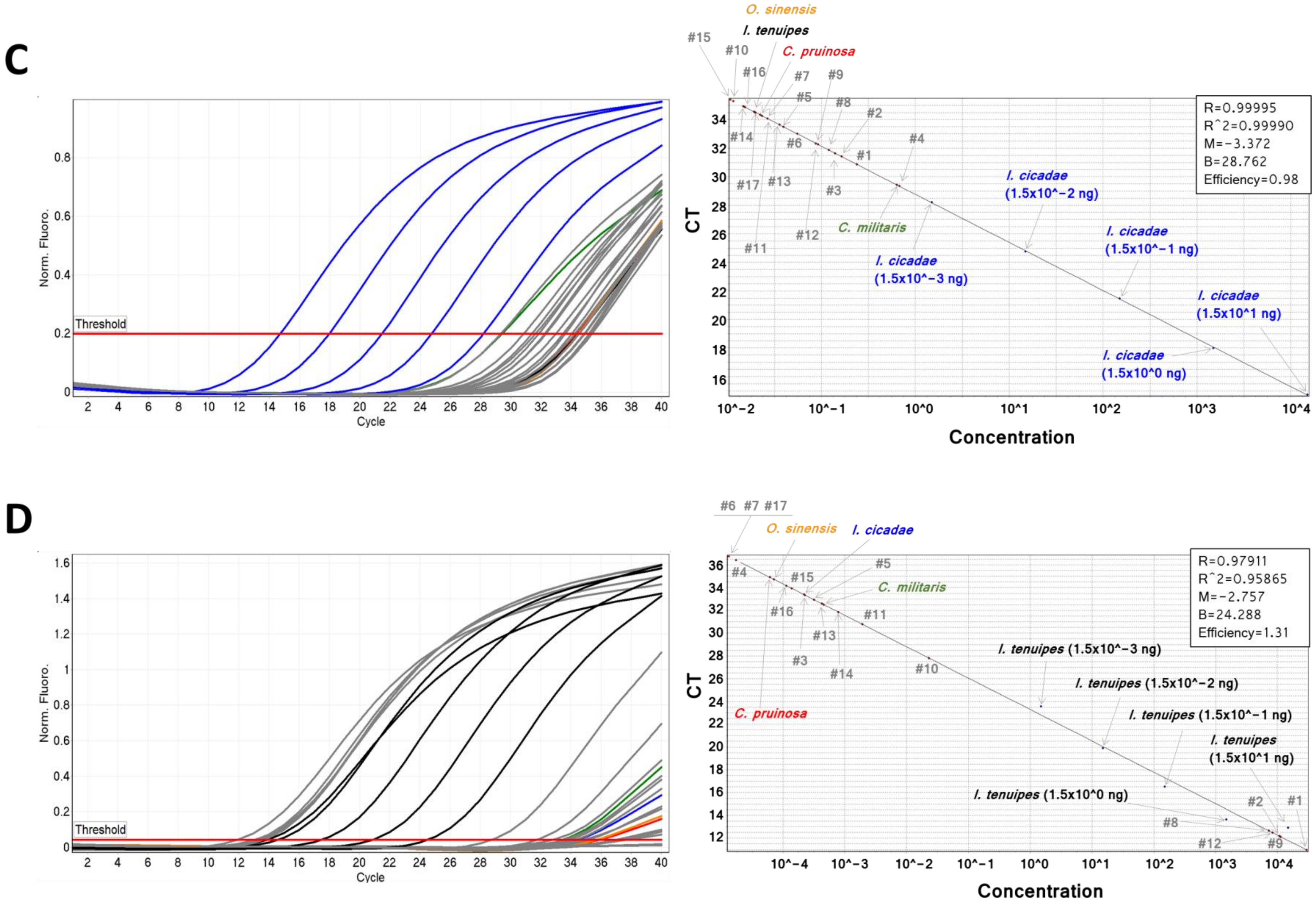
C
Threshold
Norm. Fluoro.
Cycle
O. sinensis
I. tenuipes
C. pruinosa
C. militaris
#15
#10
#16
#7
#9
#5
#8
#2
#14
#6
#4
#1
#17
#13
#3
#12
#11
I. cicadae (1.5x10^−2 ng)
I. cicadae (1.5x10^−1 ng)
I. cicadae (1.5x10^−3 ng)
I. cicadae (1.5x10^1 ng)
I. cicadae (1.5x10^0 ng)
R=0.99995
R^2=0.99990
M=−3.372
B=28.762
Efficiency=0.98
CT
Concentration
D
Threshold
Norm. Fluoro.
Cycle
#6 #7 #17
O. sinensis
I. cicadae
#4
#15
#5
C. militaris
#16
#11
#13
#3
#14
#10
C. pruinosa
I. tenuipes (1.5x10^−3 ng)
I. tenuipes (1.5x10^−2 ng)
I. tenuipes (1.5x10^−1 ng)
I. tenuipes (1.5x10^1 ng)
I. tenuipes (1.5x10^0 ng)
#2
#1
#8
#12
#9
R=0.97911
R^2=0.95865
M=−2.757
B=24.288
Efficiency=1.31
CT
Concentration

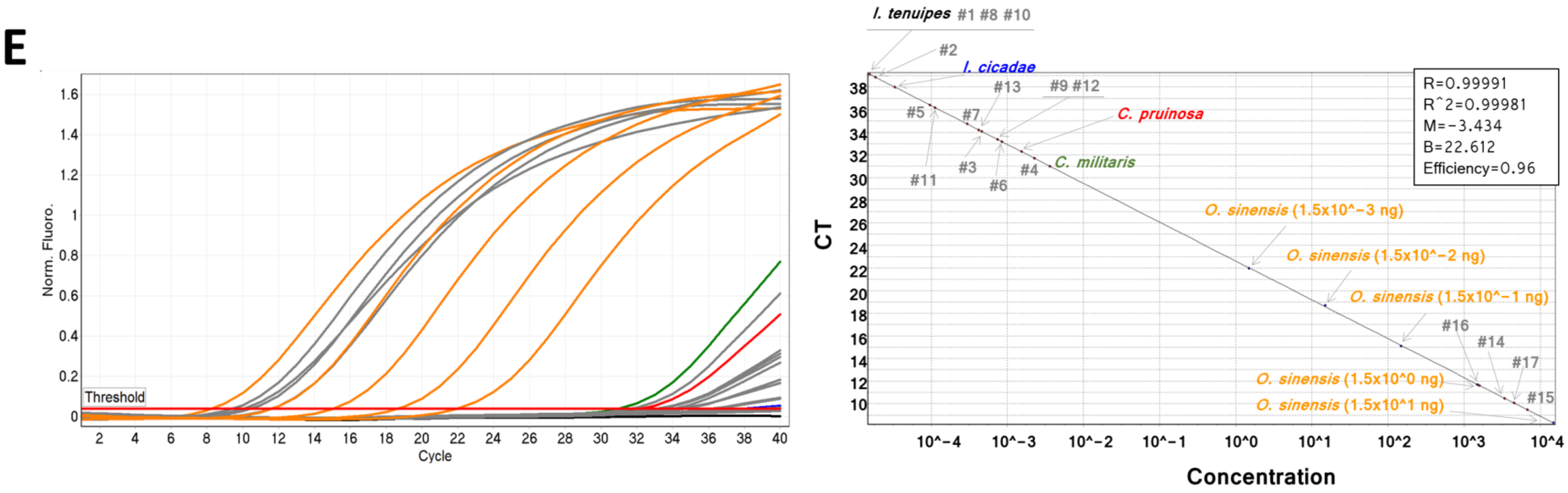

Figure S3. Establishment of the real-time PCR assay for distinguishing Cordyceps and the related species, and the results from real-time PCR assays of commercial products. (**A**) *C. militaris*, (**B**) *C. pruinosa*, (**C**) *I. cicadae*, (**D**) *I. tenuipes*, and (**E**) *O. sinensis*. Right panels: Real-time PCR data for five serial dilutions of the control genomic DNA template (15 ng, 1.5 ng, 150 pg, 15 pg, and 1.5 pg) and commercial products. Left panels: Quantification data for five serial dilutions of the control genomic DNA and commercial product templates.

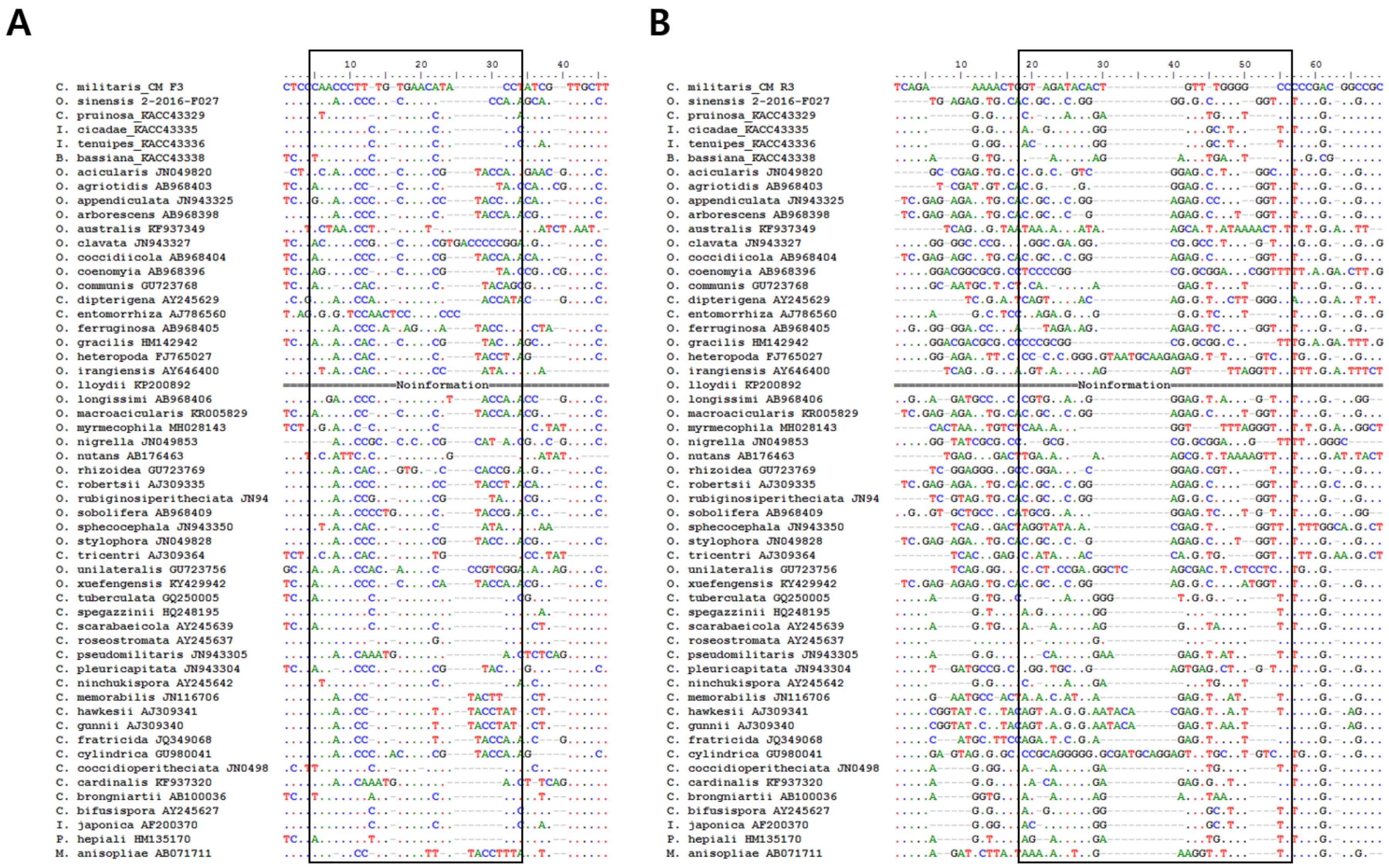
A
B

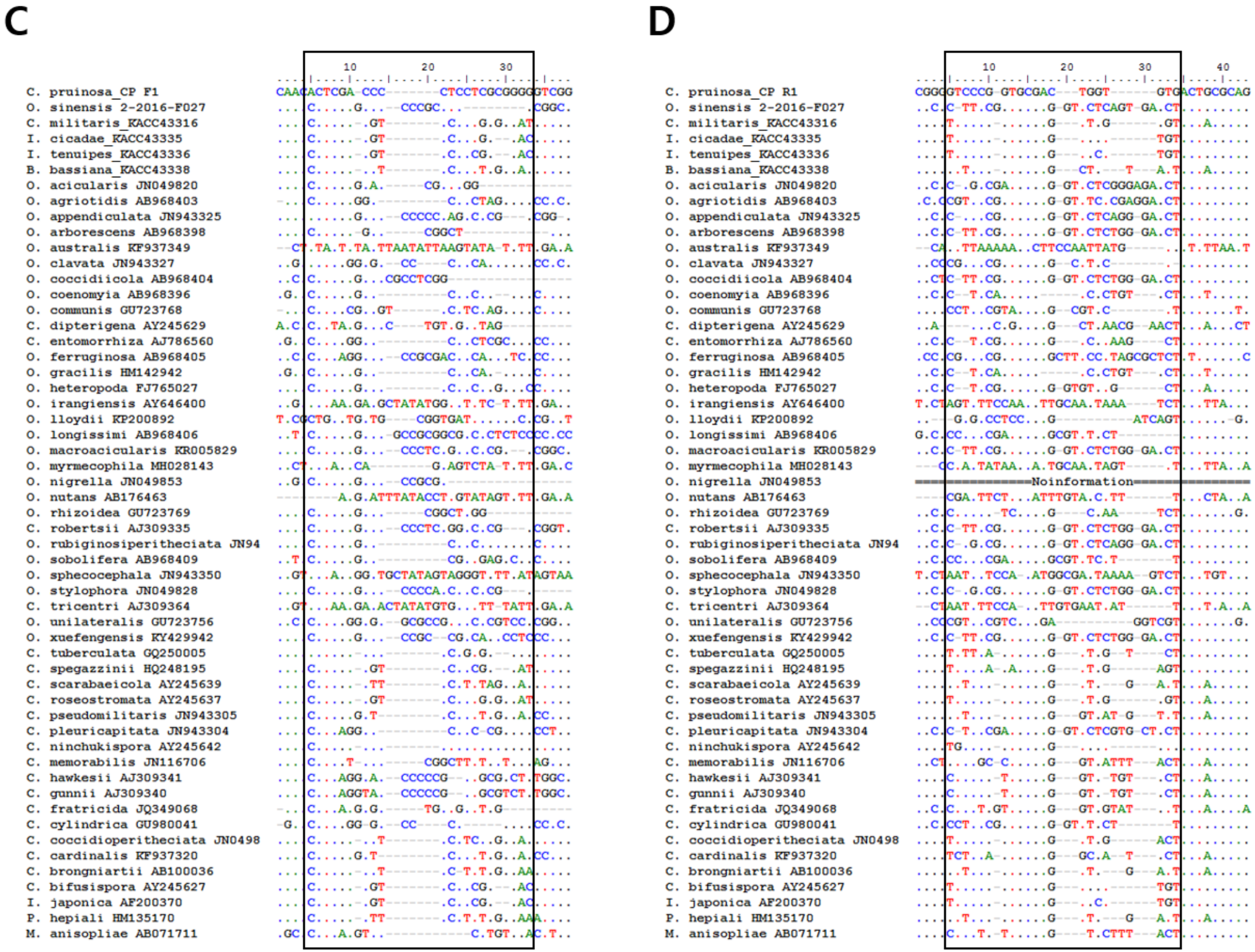
C
10
20
30
D
10
20
30
40

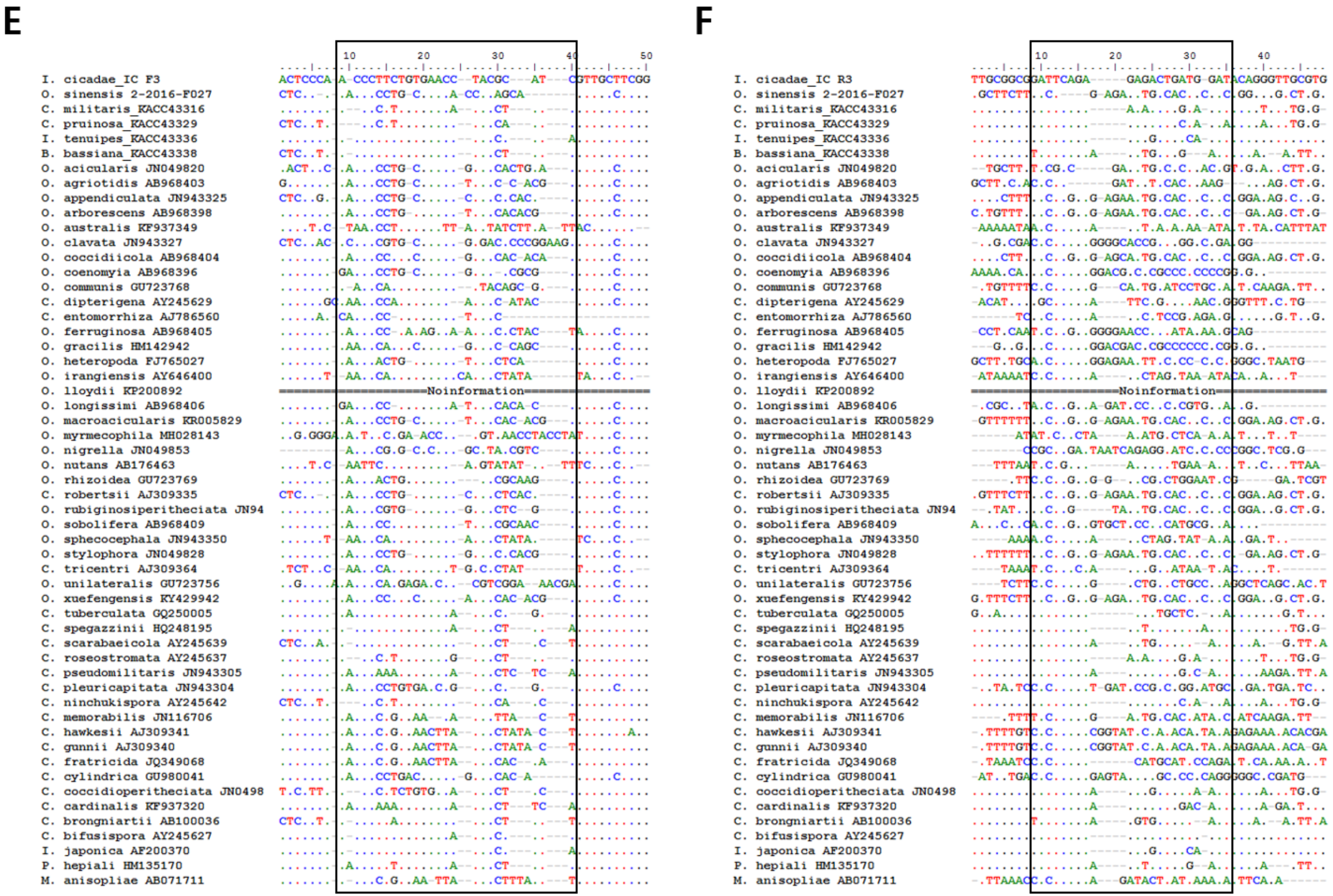
E
F

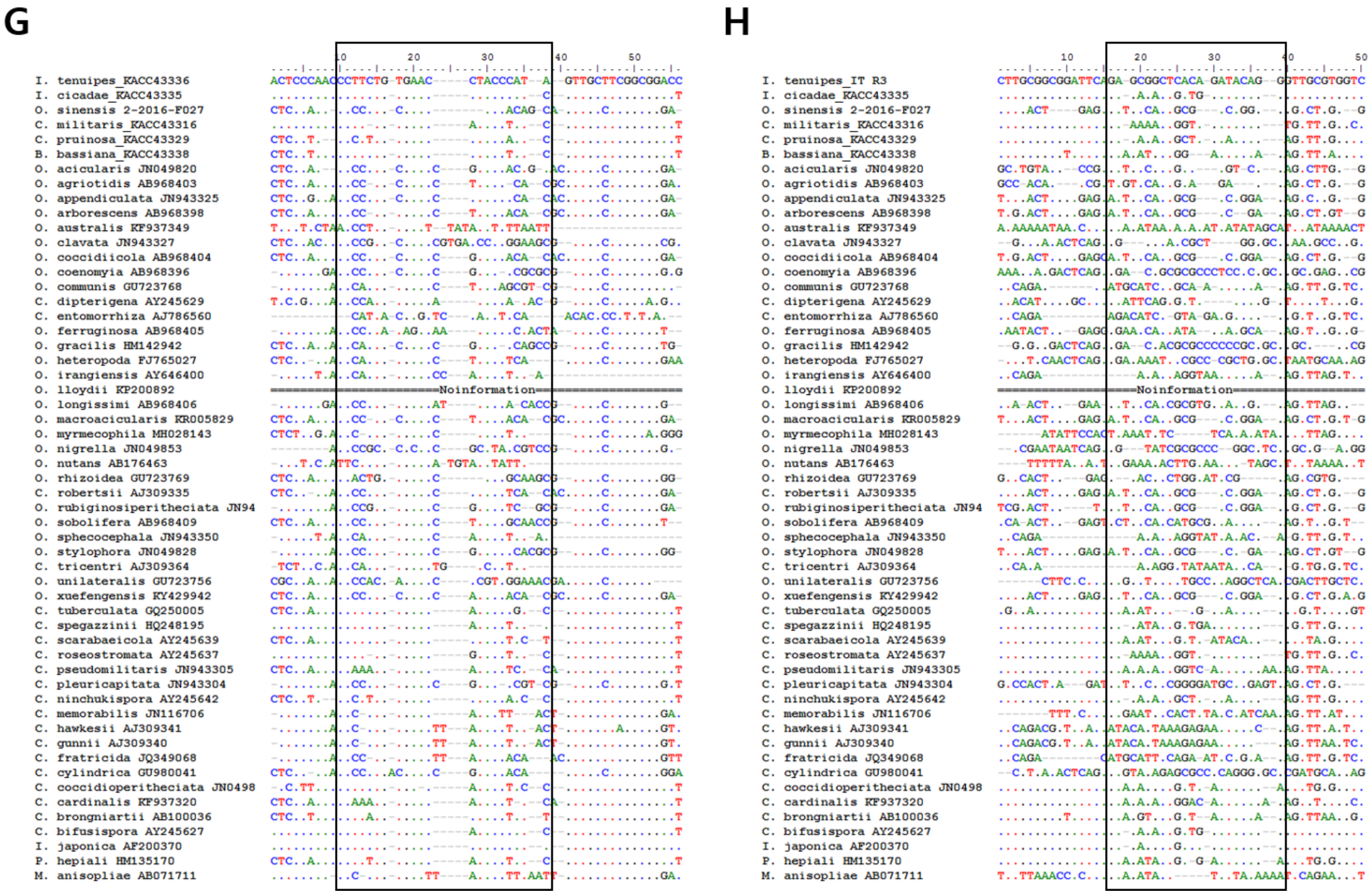
G
H
Noinformation

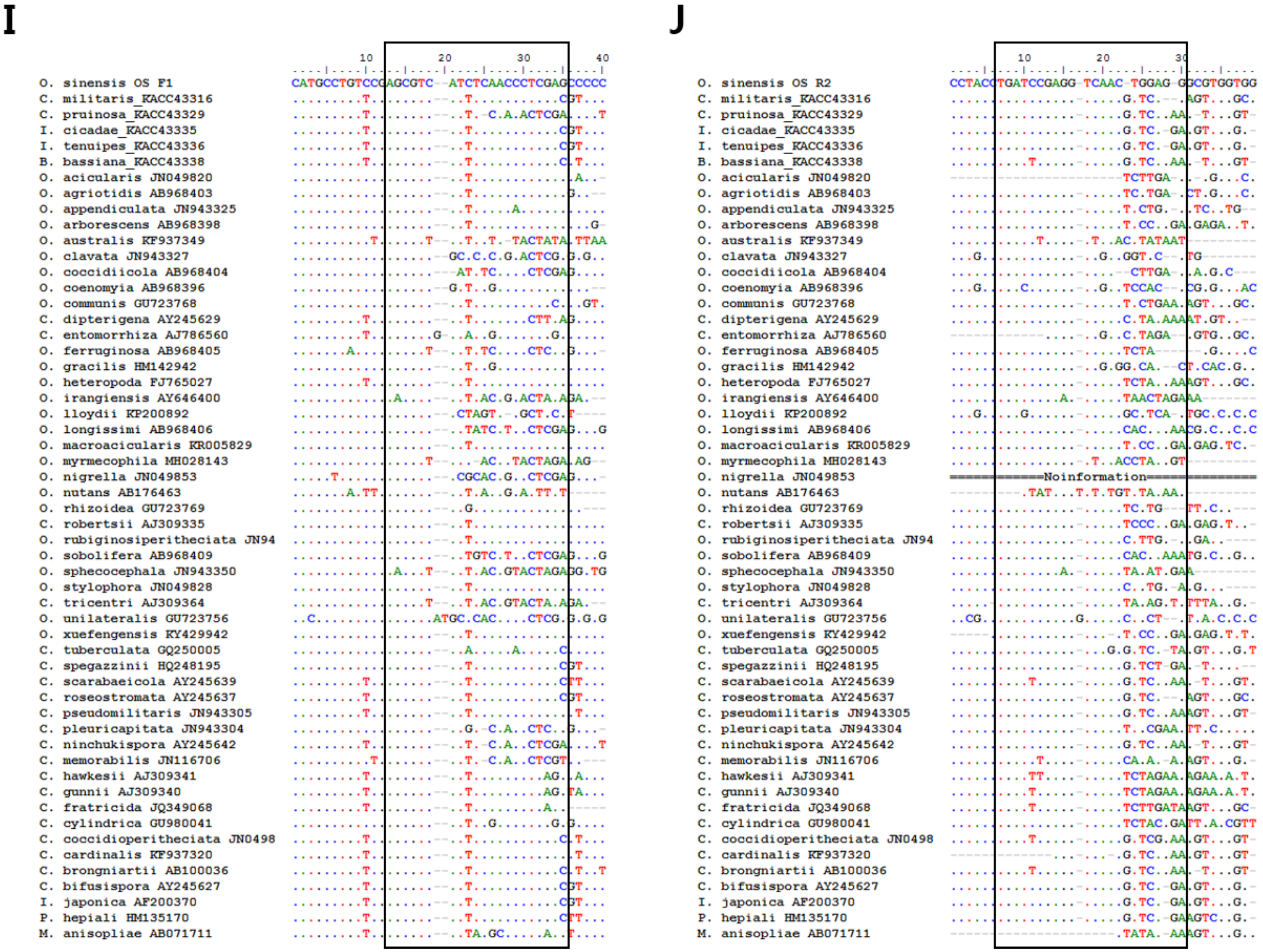
I
J
O. sinensis OS F1
C. militaris_KACC43316
C. pruinosa_KACC43329
I. cicadae_KACC43335
I. tenuipes_KACC43336
B. bassiana_KACC43338
O. acicularis JN049820
O. agriotidis AB968403
O. appendiculata JN943325
O. arborescens AB968398
O. australis KF937349
O. clavata JN943327
O. coccidiicola AB968404
O. coenomyia AB968396
O. communis GU723768
C. dipterigena AY245629
C. entomorrhiza AJ786560
O. ferruginosa AB968405
O. gracilis HM142942
O. heteropoda FJ765027
O. irangiensis AY646400
O. lloydii KP200892
O. longissimi AB968406
O. macroacicularis KR005829
O. myrmecophila MH028143
O. nigrella JN049853
O. nutans AB176463
O. rhizoidea GU723769
C. robertsii AJ309335
O. rubiginosiperitheciata JN94
O. sobolifera AB968409
O. sphecocephala JN943350
O. stylophora JN049828
C. tricentri AJ309364
O. unilateralis GU723756
O. xuefengensis KY429942
C. tuberculata GQ250005
C. spegazzinii HQ248195
C. scarabaeicola AY245639
C. roseostromata AY245637
C. pseudomilitaris JN943305
C. pleuricapitata JN943304
C. ninchukispora AY245642
C. memorabilis JN116706
C. hawkesii AJ309341
C. gunnii AJ309340
C. fratricida JQ349068
C. cylindrica GU980041
C. coccidioperitheciata JN0498
C. cardinalis KF937320
C. brongniartii AB100036
C. bifusispora AY245627
I. japonica AF200370
P. hepiali HM135170
M. anisopliae AB071711
O. sinensis OS R2
Noinformation

Figure S4. Confirmation of primer specificity by the alignment of specie-specific SCAR primer regions used for both conventional and real-time PCR assay. (**A**) Primer CM F3, (**B**) Primer CM R3, (**C**) Primer CP F1, (**D**) Primer CP R1, (**E**) Primer IC F3, **(F)** Primer IC R3, **(G)** Primer IT F4, **(H)** Primer IT R3, **(I)** Primer OS F1, and **(J)** Primer OS R2. The boxes indicate primer sequences. Dots (.) and dashes (-) indicate identical nucleotides and gaps, respectively, when compared with corresponding primer region, to maximize sequence alignment presentation.
